# Reconstructive complications and early toxicity in breast cancer patients treated with proton-based postmastectomy radiation therapy

**DOI:** 10.3389/fonc.2023.1067500

**Published:** 2023-01-20

**Authors:** Mutlay Sayan, Lara Hathout, Sarah S. Kilic, Imraan Jan, Ambroise Gilles, Natalie Hassell, Maria Kowzun, Mridula George, Lindsay Potdevin, Shicha Kumar, Jeremy Sinkin, Richard Agag, Bruce G. Haffty, Nisha Ohri

**Affiliations:** ^1^Department of Radiation Oncology, Dana-Farber Cancer Institute and Harvard Medical School, Boston, MA, United States; ^2^Department of Radiation Oncology, Brigham and Women’s Hospital and Harvard Medical School, Boston, MA, United States; ^3^Department of Radiation Oncology, Rutgers Cancer Institute of New Jersey, Rutgers University, New Brunswick, NJ, United States; ^4^Department of Radiation Oncology, Taussig Cancer Institute, Cleveland Clinic, Cleveland, OH, United States; ^5^Division of Plastic Surgery, Departments of Surgery, Rutgers Cancer Institute of New Jersey, Rutgers University, New Brunswick, NJ, United States; ^6^Departments of Surgical Oncology, Rutgers Cancer Institute of New Jersey, Rutgers University, New Brunswick, NJ, United States; ^7^Departments of Medicine, Rutgers Cancer Institute of New Jersey, Rutgers University, New Brunswick, NJ, United States

**Keywords:** carcinoma, mastectomy, proton, radiotherapy, breast

## Abstract

**Background:**

Postmastectomy radiation therapy (PMRT) decreases the risk of locoregional recurrence and increases overall survival rates in patients with high-risk node positive breast cancer. While the number of breast cancer patients treated with proton-based PMRT has increased in recent years, there is limited data on the use of proton therapy in the postmastectomy with reconstruction setting. In this study, we compared acute toxicities and reconstructive complications in patients treated with proton-based and photon-based PMRT.

**Methods:**

A retrospective review of our institutional database was performed to identify breast cancer patients treated with mastectomy with implant or autologous reconstruction followed by PMRT from 2015 to 2020. Baseline clinical, disease, and treatment related factors were compared between the photon-based and proton-based PMRT groups. Early toxicity outcomes and reconstructive complications following PMRT were graded by the treating physician.

**Results:**

A total of 11 patients treated with proton-based PMRT and 26 patients treated with photon-based PMRT were included with a median follow-up of 7.4 months (range, 0.7-33 months). Six patients (55%) in the proton group had a history of breast cancer (3 ipsilateral and 3 contralateral) and received previous RT 38 months ago (median, range 7-85). There was no significant difference in mean PMRT (p = 0.064) and boost dose (p = 0.608) between the two groups. Grade 2 skin toxicity was the most common acute toxicity in both groups (55% and 73% in the proton and photon group, respectively) (p = 0.077). Three patients (27%) in the proton group developed grade 3 skin toxicity. No Grade 4 acute toxicity was reported in either group. Reconstructive complications occurred in 4 patients (36%) in the proton group and 8 patients (31%) in photon group (p = 0.946).

**Conclusions:**

Acute skin toxicity remains the most frequent adverse event in both proton- and photon-based PMRT. In our study, reconstructive complications were not significantly higher in patients treated with proton- versus photon-based PMRT. Longer follow-up is warranted to assess late toxicities.

## Introduction

The clinical indication for proton-based radiation therapy (RT) continues to grow for treatment of various cancers. This is mainly due to the dosimetric benefits of proton-based RT which includes a low to medium entrance dose and homogenous dose distribution within the target (1–3). In addition, protons have a steep fall-off to zero dose distally to the target, known as Bragg peak, resulting in a significant normal tissue sparing, which may potentially decrease the risk of toxicity. Although these unique characteristics of protons support its use, the clinical significance of proton-based RT has not been clearly demonstrated in breast cancer patients. The RADCOMP trial is currently investigating the efficacy and cardiovascular benefits of proton-based RT in patients with non-metastatic breast cancer.

Adjuvant RT is an important component in the multidisciplinary management of breast cancer patients. In the setting of post-mastectomy, patients with high-risk node positive breast cancer often receive adjuvant RT. Postmastectomy radiation therapy (PMRT) decreases the risk of locoregional recurrence and increases overall survival rates in patients with locally advanced disease as demonstrated in multiple randomized trials, as well as the Early Breast Cancer Trialists’ Collaborative Group (EBCTCG) meta-analysis (4–7). More recently, with the increase in number of proton centers, the number of breast cancer patients treated with proton-based PMRT has increased. However, there is limited data on the use of proton therapy in the postmastectomy with reconstruction setting.

Given its significant impact on quality of life, identifying the risk factors for acute toxicities and reconstruction outcomes after PMRT is crucial. In this study, we compared acute toxicities and reconstructive complications in postmastectomy patients with implant or autologous reconstruction treated with proton-based PMRT and photon-based PMRT.

## Materials and methods

A retrospective review of our institutional database was performed to identify breast cancer patients treated with adjuvant proton or photon therapy. Patients were eligible for this study if they had mastectomy with implant or autologous reconstruction and underwent radiation therapy. Patients who underwent breast-conserving surgery and those who had mastectomy without reconstruction were excluded.

Baseline clinical characteristics were collected and included patient age, race, body mass index (BMI), history of smoking, diabetes, history of prior breast cancer and treatment received. Disease-related characteristics including histology, hormone receptor status, AJCC T stage, and AJCC N stage were also recorded. Treatment related factors included type of breast reconstruction (implant vs. autologous), receipt of chemotherapy (neoadjuvant vs. adjuvant chemotherapy), hormonal therapy, and adjuvant radiation therapy (proton- vs. photon-based PMRT).

Early toxicity outcomes (fatigue, dermatitis, pain, and esophagitis) were graded by the treating physician during the treatment course using the National Cancer Institute Common Terminology Criteria for Adverse Events (CTCAE), version 3.0. Minor reconstruction complications included infection resolved with oral antibiotics, Baker grade ≤2 capsular contracture, or fat necrosis without need for revision. Major reconstruction complications included infection or exposure of implant requiring IV antibiotics and/or operation, Baker grade ≥3 capsular contracture or fat necrosis requiring revision.

Baseline characteristics between the two groups were compared using the Chi-squared or Fisher exact test for categorical variables and a t-test for metric variables. Statistical analyses were performed using SPSS statistical software version 25 (IBM Corp., Armonk, NY, USA).

## Results

Eleven patients treated with proton therapy and 26 patients treated with photon therapy were included. Baseline patient characteristics are shown in **Table 1**. Median follow-up was 7.4 months (range, 0.7-33 months). There was no significant difference in age (p = 0.973), race (p = 0.405), laterality (p = 0.389), histology (p = 0.118), BMI ≥30 (p = 0.583), history of smoking (p = 0.228) and diabetes (p = 0.348) between the two groups. Six patients (55%) in the proton group had a history of breast cancer, of which 3 had ipsilateral and 3 had contralateral disease treated with radiation. For the three patients with a history of prior ipsilateral radiation therapy, all three patients had implant reconstruction in place at the time of proton radiation therapy. Each patient was treated with conventional fractionation in 1.8 Gy per fraction to a total dose of 4,320 to 5,750 cGy. The chest wall and axilla were treated in all three patients. The patient treated to 5,750 cGy had mild late contracture; the other two patients had no late toxicity after proton radiation therapy. The median time between previous radiation and second course of RT was 38 months (range 7-85).

**Table 1:** Baseline characteristics.

	Proton	Photon	p
Patient, n	11	26	
Age at diagnosis			
Mean, years	49.3	49.2	0.973
Race, n (%)			0.405
White	10 (91)	19 (73)	
Black or African American	1 (9)	4 (15)	
Asian	0	3 (12)	
Breast laterality, n (%)			0.389
Left	8 (73)	15 (58)	
Right	3 (27)	11 (42)	
Histology, n (%)			0.118
Invasive ductal carcinoma	11 (100)	21 (82)	
Invasive lobular carcinoma	0	5 (18)	
AJCC clinical T stage, n (%)			0.472
T1	2 (18)	4 (15)	
T2	6 (55)	8 (31)	
T3	2 (18)	11 (42)	
T4	1 (9)	3 (12)	
AJCC clinical N stage, n (%)			0.183
N0	3 (27)	2 (8)	
N1	4 (37)	15 (58)	
N2	3 (27)	3 (12)	
N3	1 (9)	6 (22)	
Receptor status, n (%)			
Estrogen receptor-positive	5 (45)	16 (62)	0.367
Progesterone receptor-positive	5 (45)	17 (65)	0.259
HER2/neu-amplified	3 (27)	6 (22)	0.267
History of smoking, n (%)	2 (18)	10 (38)	0.228
Diabetes, n (%)	2 (18)	2 (8)	0.348
BMI ≥30, n (%)	4 (37)	12 (46)	0.583
History of breast cancer, n (%)	6 (55)	0	<0.001
Ipsilateral, n (%)	3 (27)		
Treated with radiotherapy, n (%)	6 (55)	N/A	
Time since completion of radiotherapy			
Median, months (range)	38 (6.7-85.2)	N/A	
Follow-up			0.342
Median, months (range)	9.1 (2-29)	6.7 (0.7-33)	

BMI, Body mass index.

There was no difference in reconstruction types between the groups (p = 0.786). Treatment-related characteristics are shown in **Table 2**. The most common reconstruction type was implant (73% in proton and 77% in photon group). There was no significant difference in the mean PMRT dose (4797 cGy and 4986 cGy, p = 0.064) and boost dose (948 cGy vs 1033 cGy, p = 0.608) in the proton vs photon groups, respectively.

**Table 2:** Treatment‐related characteristics.

	Proton (n:11)	Photon (n:26)	*p*
**Type of breast reconstruction, n (%)**			0.786
Implant	8 (73)	20 (77)	
Autologous	3 (27)	6 (23)	
**Systemic therapy**			
Chemotherapy^a^			0.033
Neoadjuvant	3 (27)	18 (69)	
Adjuvant	8 (73)	7 (27)	
Trastuzumab	4 (36)	5 (19)	0.226
Endocrine therapy	4 (36)	18 (69)	0.063
**Radiation therapy parameters**			
Mean dose (cGy)	4797	4986	0.064
Fraction number, mean	25	25	0.668
Boost, n (%)	4 (36)	18 (69)	0.063
Mean dose (cGy)	947.5	1033.3	0.608
Radiation Field Design			0.005
3-4 fields^b^	7 (64)	26 (100)	

a One patient in photon group did not receive chemotherapy.

b Supraclavicular field with or without a posterior axillary boost.

Treatment related toxicities are shown in **Table 3**. Grade 2 skin toxicity was the most common acute toxicity in both groups (55% in proton and 73% in photon) (p = 0.077). Three patients (27%) in the proton group developed Grade 3 skin toxicity. Grade 2-3 pain was reported by 45% of patients in the proton group while only Grade 2 pain was reported by 27% patients in the photon group (p = 0.077). Grade 2 fatigue was significantly higher in the proton group (45% vs 12%, p = 0.035). No Grade 4 acute toxicity was reported in either group. There was no significant difference in reconstructive complications between the groups (36% in proton vs 31% photon group, p = 0.946).

**Table 3:** Treatment related toxicities.

	Proton (n:11)	Photon (n:26)	p
**Dermatitis, n (%)**			0.077
Grade 2	6 (55)	19 (73)	
Grade 3	3 (27)	0	
**Pain, n (%)**			
Grade 2	3 (27)	7 (27)	0.077
Grade 3	2 (18)	0	
**Fatigue, n (%)**			
Grade 2	5 (45)	3 (12)	0.035
**Esophagitis, n (%)**			
Grade 2	1 (9)	0	
**Reconstructive complication, n (%)**			0.946
Minor^a^	1 (9)	2 (8)	
Major^b^	3 (27)	6 (23)	

a Minor = infection resolved with po antibiotic, Baker grade ≤2 capsular contracture, or fat necrosis without need for revision.

b Major = infection or exposure of implant requiring IV antibiotic and/or operation, Baker grade ≥3 capsular contracture or fat necrosis require revision.

## Discussion

Within a cohort of breast cancer patients treated with proton-based PMRT, we noted acceptable rates of acute toxicities and reconstructive complications that are not significantly higher compared to patients treated with photon-based PMRT.

Evaluation of the success of the breast reconstruction following PMRT is an important aspect of the treatment outcome. Photon-based PMRT to the reconstructed breast has a complication rate of 35%, including a Baker III or IV contracture rate of 38% in implant-based reconstructions (8, 9). Consensus guidelines regarding radiation therapy in the context of breast reconstruction provide important guidance for radiation oncologists (10). However, the data on reconstructive complications following proton-based PMRT, the subject of the present study, remains limited. Several prior small studies have investigated this topic. In a small study by Luo et al. including 27 patients treated with proton-based PMRT, reconstructive complications occurred in 27% including six patients with capsular contractures and one patient with implant infection (11). Similarly, Smith et al. reported a 39% reconstruction complication rate in 42 patients following proton-based PMRT (12). In a recent study by Naoum et al. proton-based PMRT significantly increased overall reconstruction failure when compared photon-based PMRT (53% vs 43%, p-value = 0.004) (13). In our study, the reconstruction complication rate was 36% following proton-based PMRT, which is comparable to the rates reported in prior studies, and not significantly different when compared to patients treated with photon-based PMRT.

A better understanding of the risk of complications might impact the decision on when to use proton-based therapy. Similar to the incidence of reconstruction complications, no significant difference in acute skin toxicity and pain was noted between the treatment groups. However, grade 3 skin toxicity was only reported in patients treated with proton-based PMRT. This is likely due to the increased skin surface dose with proton therapy. Better understanding the proton treatment planning and improvement in treatment delivery techniques such as pencil beam versus scattered beams can help improve the acute toxicity outcomes.

Limitations of our study include its small sample size, retrospective design, and inherent confounding factors that cannot be completely accounted for in a non-randomized study. In addition, another limitation is the lack of assessment of the cosmetic outcome which is an important part of the treatment success. An additional limitation is that the small sample size of the present study limited any meaningful analysis of the impact of implant size on toxicity. Furthermore, the definition of reconstruction complication and assessment is not universally agreed upon which limits the external validity when results are compared with published studies.

In patients with a history of thoracic irradiation, challenging anatomies, or left-sided disease requiring nodal irradiation, proton therapy can provide significant advantages. However, the benefits of proton-based PMRT must be weighed against its potential complications.

## Conclusions

Our study reported similar rates of reconstructive complications and physician-reported toxicity in patients treated with proton-based and photon-based PMRT. Grade 3 skin toxicity was higher in the proton-based PMRT group. Despite being well tolerated, the benefit of proton-based PMRT is still investigational. For patients with left-side breast disease, challenging anatomies or history of previous radiation, proton-based PMRT is beneficial based on small retrospective studies while awaiting the results of the RADCOMP trial which will provide a better insight into the clinical benefits of proton therapy.

## Data availability statement

The original contributions presented in the study are included in the article/supplementary material. Further inquiries can be directed to the corresponding author.

## Ethics statement

The studies involving human participants were reviewed and approved by Rutgers Cancer Institute of New Jersey. Written informed consent for participation was not required for this study in accordance with the national legislation and the institutional requirements.

## Author contributions

All authors listed have made a substantial, direct and intellectual contribution to the work, and approved it for publication.

## References

[1] Pearlstein KA Chen RC. Comparing dosimetric, morbidity, quality of life, and cancer control outcomes after 3D conformal, intensity-modulated, and proton radiation therapy for prostate cancer. Semin Radiat Oncol (2013) 23(3):182–90. doi: 10.1016/j.semradonc.2013.01.004 23763884

[2] Bekelman JE Lu H Pugh S Baker K Berg CD Berrington de González A. Pragmatic randomised clinical trial of proton versus photon therapy for patients with non-metastatic breast cancer: the radiotherapy comparative effectiveness (RadComp) consortium trial protocol. BMJ Open (2019) 9(10):e025556. doi: 10.1136/bmjopen-2018-025556 PMC6797426 31619413

[3] Prasanna PG Rawojc K Guha C Buchsbaum JC Miszczyk JU Coleman CN. Normal tissue injury induced by photon and proton therapies: Gaps and opportunities. Int J Radiat Oncol Biol Phys (2021) 110(5):1325–40. doi: 10.1016/j.ijrobp.2021.02.043 PMC8496269 33640423

[4] Overgaard M Hansen PS Overgaard J Rose C Andersson M Bach F. Postoperative radiotherapy in high-risk premenopausal women with breast cancer who receive adjuvant chemotherapy. Danish breast cancer cooperative group 82b trial. N Engl J Med (1997) 337(14):949–55. doi: 10.1056/NEJM199710023371401 9395428

[5] Overgaard M Jensen MB Overgaard J Hansen PS Rose C Andersson M. Postoperative radiotherapy in high-risk postmenopausal breast-cancer patients given adjuvant tamoxifen: Danish breast cancer cooperative group DBCG 82c randomised trial. Lancet (1999) 353(9165):1641–8. doi: 10.1016/S0140-6736(98)09201-0 10335782

[6] Felsten G Wilcox K. Influences of stress and situation-specific mastery beliefs and satisfaction with social support on well-being and academic performance. Psychol Rep (1992) 70(1):291–303. doi: 10.2466/pr0.1992.70.1.291 1565734

[7] Clarke M Collins R Darby S Davies C Elphinstone P Evans V. Effects of radiotherapy and of differences in the extent of surgery for early breast cancer on local recurrence and 15-year survival: An overview of the randomised trials. Lancet (2005) 366(9503):2087–106. doi: 10.1016/S0140-6736(05)67887-7 16360786

[8] Yun JH Diaz R Orman AG. Breast reconstruction and radiation therapy. Cancer Control (2018) 25(1):1073274818795489. doi: 10.1177/1073274818795489 30132338 PMC6108018

[9] El-Sabawi B Sosin M Carey JN Nahabedian MY Patel KM. Breast reconstruction and adjuvant therapy: A systematic review of surgical outcomes. J Surg Oncol (2015) 112(5):458–64. doi: 10.1002/jso.24028 26345465

[10] Meattini I Becherini C Bernini M Bonzano E Criscitiello C De Rose F. Breast reconstruction and radiation therapy: An Italian expert Delphi consensus statements and critical review. Cancer Treat Rev (2021) 99:102236. doi: 10.1016/j.ctrv.2021.102236 34126314

[11] Luo L Cuaron J Braunstein L Gillespie E Kahn A McCormick B. Early outcomes of breast cancer patients treated with post-mastectomy uniform scanning proton therapy. Radiother Oncol (2019) 132:250–6. doi: 10.1016/j.radonc.2018.10.002 30414757

[12] Smith NL Jethwa KR Viehman JK Harmsen WS Gonuguntla K Elswick SM. Post-mastectomy intensity modulated proton therapy after immediate breast reconstruction: Initial report of reconstruction outcomes and predictors of complications. Radiother Oncol (2019) 140:76–83. doi: 10.1016/j.radonc.2019.05.022 31185327 PMC7734651

[13] Naoum GE Ioakeim MI Shui AM Salama L Colwell A Ho AY. Radiation modality (Proton/Photon), timing, and complication rates in patients with breast cancer receiving 2-stages Expander/Implant reconstruction. Pract Radiat Oncol (2022) 12 (6) :475–86. doi: 10.1016/j.prro.2022.05.017 PMC9637758 35989216

